# Factors Related to Greater Functional Recovery after Suffering a Stroke †

**DOI:** 10.3390/brainsci11060802

**Published:** 2021-06-17

**Authors:** María Vázquez-Guimaraens, José L. Caamaño-Ponte, Teresa Seoane-Pillado, Javier Cudeiro

**Affiliations:** 1 Hospital Universitario de A Coruña, Servizo Galego de Saúde, 15009 A Coruña, Spain; 2 CTX a Veiga (Láncara), 27360 Lugo, Spain; caamajos@hotmail.com; 3 Universitat Oberta de Catalunya, 08018 Barcelona, Spain; 4 Área de Medicina Preventiva y Salud Pública, Departamento de Ciencias de la Salud, Universidad de A Coruña–INIBIC, 15403 A Coruña, Spain; maria.teresa.seoane.pillado@udc.es; 5 Neuroscience and Motor Control Group (NEUROcom)-Instituto Biomédico de A Coruña (INIBIC), Universidad de A Coruña, 15006 Oza, Spain; javier.cudeiro@udc.es; 6 Centro de Estimulación Cerebral de Galicia, 15009 A Coruña, Spain

**Keywords:** stroke, rehabilitation, functional independence measure, recovery, functionality, predictors of outcome, cerebrovascular accident, neurorehabilitation, degree of disability

## Abstract

Background: In a stroke, the importance of initial functional status is fundamental for prognosis. The aim of the current study was to investigate functional status, assessed by the Functional Independence Measure (FIM) scale, and possible predictors of functional outcome at discharge from inpatient rehabilitation. Methods: This is a retrospective study that was carried out at the Physical Medicine and Rehabilitation Service in A Coruña (Spain). A total of 365 consecutive patients with primary diagnosis of stroke were enrolled. The functional assessments of all patients were performed through the FIM. A descriptive and a bivariate analysis of the variables included in the study was made and a succession of linear regression models was used to determine which variables were associated with the total FIM at discharge. Results: Prior to having the stroke, 76.7% were totally independent in activities of daily living. The FIM scale score was 52.5 ± 25.5 points at admission and 83.4 ± 26.3 at hospital discharge. The multivariate analysis showed that FIM scores on admission were the most important predictors of FIM outcomes. Conclusions: Our study indicates that the degree of independence prior to admission after suffering a stroke is the factor that will determine the functionality of patients at hospital discharge.

## 1. Introduction

Cerebrovascular accident (CVA) or stroke is one of the leading causes of mortality in the Western world, and the leading cause of permanent disability in adulthood [1]. Its high prevalence produces a great impact on our society and it is estimated that in the coming years, it will increase more, which will generate a greater number of people in need of care [1].

The majority of studies suggest that neurological recovery occurs in the first three months with maximum recovery estimated in the first 4–6 weeks after the stroke [2,3]. It is important to point out that the therapeutic process in stroke patients calls for a multidisciplinary approach addressing the deficit caused, and the physician specializing in physical medicine and rehabilitation plays and important role in evaluating, coordinating and planning the needs of each patient in an individualized way. The objective of the rehabilitation of patients who have suffered a stroke is to achieve the maximum functional and social capacity that allows them to reintegrate into their previous activities. The neurorehabilitation programs have irrefutably confirmed their effectiveness in reducing both mortality and the degree of disability and dependency [4]. The neurorehabilitation programs should be started as soon as possible once the treatment indication has been evaluated [5].

The medical literature indicates modifiable factors that irrevocably influence the functional improvement of these patients, including the time of onset of rehabilitation, the duration and intensity of the treatment, and where it is received; the treatment period should be determined individually, based on the severity of the deficits, cognitive ability and comorbidity, as well as the response to the established objectives [6,7]. In this sense, in a recent systematic review, Meyer et al. (2015) summarize the utility of variables available at acute discharge after stroke for predicting functional independence at discharge from inpatient rehabilitation [8]. The authors stated that changes in post-rehabilitation function, as measured by the BI (Barthel Index) or the FIM (Functional Independence Measure), can be explained by only a few variables, being very relevant admission functional level and also including NIHSS (National Institute of Health Stroke Scale), dysphasia, impulsivity, neglect, previous stroke, and age. At the same time, the authors encourage us to explore further the predictive utility of those variables. This is what our work intends to do.

Time spent in inpatient rehabilitation ranges from 4–12 weeks, depending on the severity of the deficits and/or the appearance of potential complications [9,10]. The results available indicate the recovery progress all along this time, and therefore, brain plasticity processes are optimized if rehabilitation programs are started early and last for at least 6 months in the most severe strokes. The studies confirm that the admission of these patients to stroke rehabilitation units decreases mortality, disability, and the need to refer to assisted living [11,12]. 

As mentioned above, our study deals with a topic that is widely discussed in the area of neurorehabilitation: Factors that will determine the functionality of patients at hospital discharge. Recent publications, such as Thorpe et al. [13], suggest that the evolution of people who survive a stroke can be defined basically by the referral of the patient to discharge (home or institutionalized care), their quality of life, or their functional disability. Whether the patient returns or not to a family home or assisted institution is an important social factor. Stein et al. established that socio-demographic characteristics and degree of dependence prior to stroke are the factors that most determine whether the patient will return to the family domicile, or need to be institutionalized after hospital discharge [14]. To assess functional capacity and independence, they used the BI; however, this scale does not take into account the cognitive component, so we decided to use a scale that includes it and that allows quantifying changes over time, such as the FIM scale.

A large majority of studies include patients with stroke that come from a specialized unit [8,10,11,14]. However, specialists in rehabilitation medicine have to be prepared to identify the factors that influence the final outcome of the patients after neurorehabilitation, use the tools usually available, and be able to develop strategies that help to improve their functional capacity and accordingly, their quality of life [15,16,17]. For this reason, this work is aimed to quantify the degree of functional disability of patients who had suffered a stroke and determine the factors that will favor recovery.

## 2. Materials and Methods

### 2.1. Study Design

A retrospective observational study was carried out: 365 patients with an acute or subacute stroke admitted between January 2010 and December 2014 were selected at our physical medicine and rehabilitation unit at the Hospital Marítimo de Oza, in A Coruña (Spain). The regional ethics committee, as required by law, approved the study; all of the patients involved in the study signed an informed consent form. The procedures that were carried out during the study are in accordance with the ethical standards of the declaration of Helsinki.

### 2.2. Sample

Inclusion criteria were: First episode of cerebrovascular accident (ischemic or hemorrhagic etiology), brain injury confirmed by image techniques, persons with a good cognitive level, and those over the age of 18. Patients with subarachnoid, subdural, or epidural hemorrhages, patients with previous neurological impairment, cognitive impairment that limited the scales of assessment, and those who had died or moved to other services during hospital admission were excluded.

The study included a total of 365 patients (107 hemorrhagic patients and 256 patients with ischemic events). This sample size makes it possible estimate the average score of the FIM scale in patients who have suffered a hemorrhagic event with a 95% confidence level and an accuracy of ±5.1, assuming that the standard deviation of the functionality scale score is approximately 27 population-level points. In the case of patients with ischemic events, 256 subjects were selected, which allows estimating the parameter of interest with 95% confidence and accuracy of ±3.3.

All of patients underwent a functional evaluation at the time of admission and on the day of their discharge from the hospital to classify their degree of disability. Variables were obtained by clinical evaluation and a subsequent interview. The evaluator (always the same) was a well-trained physician specializing in physical medicine and rehabilitation. All the subjects received daily therapy (physiotherapy and occupational therapy or speech therapy according to their needs) during their hospital stay (see Table 1 below).

### 2.3. Variables and Functional Assessment

The variables included in the present analysis were: (1) Qualitative variables: Etiology of the CVA, gender, region of residence, independence for activities of daily living prior to stroke, originating medical service, characteristics of brain injury, laterality (left, right or bilateral), rehabilitation therapies carried out (number of sessions of physiotherapy, occupational therapy and speech therapy), and destination after hospital discharge (home or institutionalized care); (2) quantitative variables: Age, length of stay, and Functional Independence Measure (FIM) scale.

The functional assessment of all patients was performed using the FIM. The FIM is the most widely accepted instrument as a measure of functionality in the field of rehabilitation. It is based on 7 levels of functionality and 18 items are defined within 6 areas, subdivided into motor FIM (personal care, sphincter control, ambulation, and mobility) and cognitive FIM (communication and social knowledge). The maximum score for each item is 7 and the minimum is 1, so the maximum total FIM is 126 points, and the minimum is 18 points [18]. The FIM is a tool used to determine functional capacity in patients admitted to rehabilitation. It discriminates patients according to age, morbidity, and destination to discharge; it has a great internal congruence, discriminatory capacity for patients in rehabilitation, makes it possible to measure changes over time, and has a good correlation with other scales. It distinguishes different degrees of severity between patients with spinal cord injury and cerebrovascular disease and is a good indicator of the amount of care, and demonstrates responses and ability to measure changes over time. In addition, it is able to assess the type of care and the amount of help a person requires to perform basic activities of daily living safely and effectively, and the impact of the disability on the patient’s life [18,19].

### 2.4. Statistical Analysis

A descriptive analysis of all the variables included in the study was carried out, expressing the categorical variables as absolute value and percentage with estimation of the 95% confidence interval, and the quantitative as mean ± standard deviation (SD), median, and range.

The association between categorical variables was studied with the chi-square test. To compare the means between ischemic strokes versus hemorrhagic strokes, the Student’s t or a non-parametric analysis (Mann–Whitney test) were performed depending on normality (Kolmogorov–Smirnov test). To compare the values of the FIM scale according to the established age groups, the Kruskall–Wallis non-parametric test was used. The association between the values of the FIM scale at admission and at the time of discharge was performed using the Spearman correlation coefficient.

Subsequently, multiple linear regression models were used to determine which variables were associated with the total FIM at discharge, adjusting for the variables that in the bivariate analysis showed association with the response. Furthermore, the variables included were those considered more important and very often found in the literature [15,16,17,18]: Etiology, sex, age, and FIM at admission. Coefficient of determination was used to select the regression model. The coefficient measures how much of the variability of the FIM values can be explained by the independent variables; the criterion was to select the prediction model with the highest coefficient of determination.

The analyses were carried out with the statistical package “Statistical Package for Social Science Software”, version 19.0 (IBM Company, Chicago, IL, USA), directly under the supervision of the statistical service (University Hospital of A Coruña). A two-tailed *p* value < 0.05 was considered to be significant.

## 3. Results

### 3.1. The Characteristics of the Sample

Prior to the stroke, 50.1% of patients lived in the urban area, 76.7% were totally independent for activities of daily living, 62.2% of the patients were males, and the mean age was 66.8 (range, 22–91) years old (see Table 1).

Depending on the etiology of the cerebrovascular accident, 70.1% of the patients were ischemic and 21.9% had aphasia when they arrived at our physical medicine and rehabilitation service. A total of 41.6% of the sample came from neurology, 9.9% from neurosurgery, 42.5% from internal medicine, and the rest (6.0%) came from other services (cardiology, surgery, pneumology, otorhinolaryngology, and urology). 

The characteristics of the brain injury were as follows: The supratentorial region was the most affected in our patients, almost equally on the right side as the left, and only 17 patients (4.7%) had bilateral lesions. 

All the patients studied underwent physiotherapy (Bobath) during their admission to the physical medicine and rehabilitation service: 75 of them had already begun physiotherapy (Bobath) during their prior admission to other units; 23.3% of the patients were undergoing occupational therapy; and 15.3% required speech therapy. 

The length of stay was 78.7 ± 49 days, and 90.7% of all patients returned to their family homes and only 9.3% had to be institutionalized in care centres.

**Table 1:** Characteristics of the sample.

		Mean ± SD	Median [Range]
Age (years)		66.8 ± 12.0	69.0 [22–91]
Length of stay (days)		78.7 ± 49.0	73.0 [3–280]
		**n [%]**	**95% CI**
Type of stroke	Ischemic	256 [70.1]	[65.3–74.9]
	Hemorrhagic	109 [29.9]	[25.0–34.7]
Gender	Male	227 [62.2]	[57.1–67.3]
	Female	138 [37.8]	[32.7–42.9]
Area	Countryside	182 [49.9]	[44.6–55.1]
	Urban	183 [50.1]	[44.9–55.4]
Activities of daily living	Independent	280 [76.7]	[72.2–81.2]
	Not independent	85 [23.3]	[18.8–27.8]
Service of origin	Neurology	152 [41.6]	[36.5–46.8]
	Others services	213 [58.4]	[53.2–63.6]
Brain injury localization	Supratentorial	311 [85.2]	[81.4–89.0]
	Infratentorial	54 [14.8]	[11.0–18.6]
Laterality	Left	176 [48.2]	[43.0–53.5]
	Right	172 [47.1]	[41.9–52.4]
	Bilateral	17 [4.7]	[2.6–6.9]
Language disorder	Dysarthria	94 [25.8]	[21.1–30.4]
	Aphasia	80 [21.9]	[17.5–26.3]
Rehabilitation treatments	Physiotherapy	365 [100.0]	[99.0–100.0]
	Occupational therapy	85 [23.3]	[18.8–27.8]
	Speech therapy	56 [15.3]	[11.5–19.2]
Destination at discharge	Family home	330 [90.7]	[87.3–93.6]
	Care centers	34 [9.3]	[6.2–12.4]

### 3.2. Functionality of the Patients

Analysis of the FIM score at discharge and its associated variables is shown in Table 2. On evaluating the correlation between the values of the FIM scale at admission and at the time of discharge, we obtained a linear coefficient of magnitude 0.741, *p* < 0.001. 

Comparing gender, men showed higher scores on the FIM scale at discharge, although no significant differences were found (84.6 ± 26.4 vs. 81.5 ± 26.0, *p* = 0.273). After evaluating the global FIM scale at discharge regarding the age of the patients, we concluded that there was no statistically significant linear correlation (r = −0.089; *p* = 0.091). If we categorize the patient’s age variable as follows: ≤60 years, 60–70 years, and >70 years, there was no significant difference either. Patients from the urban area achieved slightly greater functional independence than patients from rural areas but statistical significance was not reached (84.4 ± 26.8 vs. 82.4 ± 25.8, *p* = 0.466).

Statistically significant differences were found in the following: When we related the degree of independence before admission with functional status at discharge, we observed that those who were totally independent before CVA showed significantly higher scores than patients with some degree of dependence for activities of daily living (85.3 ± 25.5 vs. 77.3 ± 28.0, *p* = 0.014). Patients referred from the neurology service had a significantly higher mean FIM scale score than patients from other hospital services (91.2 ± 21.1 vs. 77.9 ± 28.2, *p* < 0.001). On evaluating the presence of language disorders at the time of admission, we found that patients without aphasia acquired significantly higher scores than patients with aphasia (88.0 ± 25.3 vs. 67.0 ± 23.0, *p* < 0.001). It should be noted that patients who did not require speech therapy had higher scores after hospital discharge (86.1 ± 25.7 vs. 68.9 ± 25.7, *p* < 0.001); however, there were no significant differences with respect to patients who required occupational therapy. Comparing gender, men showed higher scores on the FIM scale at discharge, although no significant differences were found (84.6 ± 26.4 vs. 81.5 ± 26.0, *p* = 0.273). After evaluating the global FIM scale at discharge regarding the age of the patients, we concluded that there was no statistically significant linear correlation (r = −0.089; *p* = 0.091). If we categorize the patient’s age variable as follows: ≤60 years, 60–70 years, and >70 years, there was no significant difference either. 

**Table 2:** Functionality of the patients. Analysis of the Functional Independence Measure score at discharge and its associated variables.

		N	Mean ± SD	*p*
Gender	Women	138	81.5 ± 26.0	0.273
	Men	227	84.6 ± 26.4	
Age	<60 years old (women:29; men: 74)	103	83.0 ± 27.8	0.053
	60–70 years old (women: 37; men: 62)	99	88.7 ± 22.4	
	>70 years (women: 72; men: 91)	163	80.5 ± 27.1	
Area	Urban	183	84.4 ± 26.8	0.466
	Countryside	182	82.4 ± 25.8	
Previously independent	Yes	280	85.3 ± 25.5	0.014
	No	85	77.3 ± 28.0	
Service of origin	Neurology	152	91.2 ± 21.1	<0.001
	Others	213	77.9 ± 28.2	
Language disorder	Aphasia	80	67.0 ± 23.0	<0.001
	Not aphasia	285	88.0 ± 25.3	
Occupational therapy	Executed	85	80.6 ± 23.5	0.266
	Not executed	280	84.3 ± 27.0	
Speech therapy	Executed	56	68.9 ± 25.7	<0.001
	Not executed	309	86.1 ± 25.7	

### 3.3. Functionality of the Sample and Functionality According to the Etiology of the Stroke

Patients with ischemic stroke had higher scores in all of the components of the FIM scale than hemorrhagic patients, with only significant differences in cognitive and total (motor and cognitive) scores on admission (16.8 ± 10.5 vs. 19.6 ± 10.0, *p* = 0.016 and 48.4 ± 25.5 vs. 54.3 ± 25.4, *p* = 0.044) (see Table 3).

No significant differences were found on the functional scale score according to the etiology of the patient; the discharge score for ischemic patients was 85.1 ± 25.1, and for hemorrhagic patients was 79.6 ± 28.6; (*p* = 0.085).

When investigating the functional improvement after the stay in the physical medicine and rehabilitation service, we observed a tendency for hemorrhagic patients to improve more than ischemic patients, but it was not significant.

**Table 3:** Functionality of the sample and functionality according to the etiology of the stroke.

	Total	Ischemic	Hemorrhagic	
FIM	Mean ± SD	Mean ± SD	Mean ± SD	*p*
Admission Motor	33.7 ± 18.1	34.6 ± 18.0	31.6 ± 18.4	0.141
Admission Cognitive	18.8 ± 10.2	19.6 ± 10.0	16.8 ± 10.5	0.016
Admission Total	52.5 ± 25.5	54.3 ± 25.4	48.4 ± 25.5	0.044
Discharge Motor	58.4 ± 19.9	59.6 ± 19.4	55.5 ± 20.9	0.071
Discharge Cognitive	25.0 ± 9.5	25.4 ± 8.1	24.1 ± 12.1	0.284
Discharge Total	83.4 ± 26.3	85.1 ± 25.1	79.6 ± 28.6	0.085
Improvement achieved	30.9 ± 19.1	30.8 ± 17.8	31.2 ± 21.8	0.856

### 3.4. Prediction of Total FIM Score at Discharge

In the multivariate linear regression model to predict the values of total FIM at discharge, it was found that the variables sex, age, etiology, and degree of independence prior to admission did not show a statistically significant association (Table 4). The total FIM score at admission was the only variable that predicted functionality at discharge from our service (*p* < 0.001).

**Table 4:** Prediction of total FIM score at discharge. β = Regression coefficient. IC 95% = Estimated confidence interval of the regression coefficient.

	Β	Std. Error	*p*	IC 95% for β
Type of stroke [ref: Ischemic]	−1.397	2.120	0.510	[−5.566; 2.772]
Gender [ref: Women]	0.595	1.962	0.762	[−3.263; 4.453]
Age	−0.64	0.082	0.439	[−0.225; 0.098]
Katz index [Independence]	−2.945	2.273	0.196	[−7.415; 1.525]
FIM on Admission	0.741	0.038	<0.001	[0.668; 0.815]

## 4. Discussion

The main outcome of our study indicates that the degree of independence before admission after suffering a stroke is the factor that will determine the functionality of the patients after hospital discharge. Therefore, FIM is a very valuable tool to predict functional improvement after neurorehabilitation since the total FIM score at admission was the only variable that predicted functionality at discharge from our study. Early identification of predictive factors relevant to functional outcomes for stroke patients is very relevant to the establishment of an effective continuing care program. Furthermore, given the importance of decisions about rehabilitation suitability, we believe that providing rehabilitation specialists with simple tools to improve their decision-making can be of great value.

A relevant strength of this manuscript is the relatively large retrospective sample that included patients with stroke admitted to a rehabilitation unit between 2010 and 2014. On the other hand, another important characteristic of our work is that it gathers the largest sample of patients in the northwest of Spain (Galicia) and includes populations of rural and urban origin in equivalent proportions. Furthermore, our sample indicates that the improvement was similar in both groups. Thus, our results can be compared to any area, both urban and rural.

The patients in our study suffered from strokes at around 68 years of age, and were predominantly male (62%), supporting the findings of other authors who found that men are more likely to have strokes than women, although they do have a higher degree of disability [20]. However, unlike in our study, in a large number of studies the stroke patients came from a specialized stroke unit (not available when our work was conducted) and the patients were referred from different services, mainly from neurology, neurosurgery, and internal medicine [21,22]. In addition, the fact that the Hospital of A Coruña does not have a geriatrics service means that a potential group of patients with strokes who are susceptible to a transverse intervention model can be referred to other services without taking into account the specific characteristics of multi-medicated patients, as they are usually people over the age of 65, and who constitute a relevant subgroup in the population that we have examined. In our sample, it should be noted that patients referred from the neurology service improved more after rehabilitation than those from other specialized departments; the type of selection process performed on neurological patients justifies the reason for this. As we did not have a stroke unit in our hospital, young patients with ischemic strokes entered the neurology service, while elderly people or those with a hemorrhagic cause were referred to other services or units. It seems logical that stays in our physical medicine and rehabilitation service were longer than in other studies in as much as we received patients early in the acute/sub-acute phase [10,23,24]. This is confirmed by analysing the significant degree of disability with which they entered our service in comparison to other published studies. Therefore, a contribution of our study is to show, on the one hand, the functional results of stroke patients treated at the physical medicine and rehabilitation service, and on the other, to report the main results of the treatment: their degree of independence and destination at discharge. Our findings support and extend previous studies on functional outcome after suffering a stroke, expanding contributions in this field, such as establishing the factors that interfere in the rehabilitation of stroke victims [25,26,27].

There is evidence that rehabilitation services help to decrease dependency, hospitalization time, and the need for institutionalization at discharge, although there are few studies available that have been carried out in our country on the outcome of patients after suffering a stroke [28,29,30,31]. A study carried out in 2014, published in the *European Journal of Physical and Rehabilitation Medicine*, indicated that 81.5% of patients returned to their households after suffering a CVA, while another study published in 2016, also with a Spanish sample, indicated a percentage of 84.8% [32,33]. In our study, we obtained more promising results, as 90.7% of patients returned to their family homes. This could be explained by the fact that a large number of patients achieved an adequate functional level to be able to adapt to their domestic environment. It was probably due to the longer stay in our hospital, likely due to the fact that we admit most patients early in the acute/subacute phase, which justifies that the stay is longer.

We observed that patients derived from the neurology service have greater functionality on completing intensive rehabilitation. This can be justified as the elderly, dependent, and hemorrhagic etiology patients in our hospital usually enter services other than the neurology service; these patients present lower scores on the FIM scale on admission, and consequently, a greater degree of disability on their arrival at our service. 

A substantial part of our work is related to the different factors that can positively affect the recovery of patients. Disagreeing with other studies, we found than being male, young, having suffered an ischemic stroke did not predict a higher score on the FIM scale at discharge [34,35,36]. Likewise, when we relate the degree of independence prior to admission with functional status at discharge, we find that patients who were totally independent achieved significantly higher scores than those that entered with some degree of dependency; however, there are very few publications on stroke that study these pre-stroke factors.

The studies of Katrak, Kelly, and Paolucci, which involved 718, 1064, and 270 patients, respectively, indicated that patients with hemorrhagic etiology made greater functional gains following their stay in the physical medicine and rehabilitation service [28,37,38]. Our studies support this trend, but since they do not reach statistical significance, it would be risky to draw relevant conclusions from these data.

Our research presents limitations that should be considered when interpreting the results we have presented here. Our findings cannot be generalized to all patients who have suffered a stroke, but they are representative of the degree of recovery achieved in those who have undergone intensive rehabilitative treatment during their admission to a Neurorehabilitation Unit in our environment. Moreover, although all the patients received physical therapy, the specific type of therapy received has not been included, so it would be interesting in future studies to include a description of the different techniques used. On the other hand, there are several strengths in our research that are worth mentioning. All the studied cases were confirmed by neuroimaging, as a result of which we consider it unlikely that there was a failed classification of the mechanism of the stroke or its precise location. Another strength of our approach is that the sample sizes are large, which provided adequate statistical power to detect clinically relevant and statistically significant differences. Because the FIM is more sensitive to detecting changes over time, its use as a measuring instrument made it possible to quantity the recovery with greater precision in terms of functional result. Finally, it is worth mentioning that our study fits very well with the results published by Lin et al. [39], which show that FIM at admission was the best predictor of FIM at discharge, although the variables included in the regression model where not the same. Lin’s study quantified stroke severity using the Canadian Neurological Scale (CNS) and we did not.

## 5. Conclusions

This work helps to expand the published data on the functionality of patients with strokes after their admission to a physical medicine and rehabilitation service specializing in neurorehabilitation. 

Our study indicates that the degree of independence prior to admission after suffering a stroke is the factor that will determine the functionality of patients at hospital discharge.

## Data Availability

The analyses were carried out with the statistical package “Statistical Package for Social Science Software”, version 19.0 (IBM Company, Chicago, IL, USA) directly under the supervision of The Statistical Service (University Hospital of A Coruña).
